# An open-source phase correction toolkit for transcranial focused ultrasound

**DOI:** 10.1186/s42490-020-00043-3

**Published:** 2020-08-14

**Authors:** Changzhu Jin, David Moore, John Snell, Dong-Guk Paeng

**Affiliations:** 1 Department of Robotics Engineering, DGIST, Daegu, 42988 Korea; 2 DGIST-ETH Microrobot Research Center, DGIST, Daegu, 42988 Korea; 3 Focused Ultrasound Foundation, Charlottesville, VA 22903 USA; 4 Department of Radiology and Medical Imaging, University of Virginia, Charlottesville, VA 22903 USA; 5 Ocean System, Engineering/ Biomedical Engineering, Jeju National University, Jeju, 63243 Korea

**Keywords:** Transcranial focused ultrasound, Ray-based phase correction, Open-source toolkit

## Abstract

**Background:**

The phase correction on transcranial focused ultrasound is essential to regulate unwanted focal point shift caused by skull bone aberration. The aim of the current study was to design and investigate the feasibility of a ray-based phase correction toolkit for transcranial focused ultrasound.

**Results:**

The peak pressure at focal area was improved by 140.5 ± 7.0% on target I and 134.8 ± 19.1% on target II using proposed phase correction toolkit, respectively. A total computation time of 402.1 ± 24.5 milliseconds was achieved for each sonication.

**Conclusion:**

The designed ray-based phase correction software can be used as a lightweight toolkit to compensate aberrated phase within clinical environment.

## Background

Transcranial focused ultrasound (tcFUS) using a large-phased array transducer has become an attractive modality to treat many brain diseases. Initial clinical trials have reported about the treatment of brain tumor [1], neuropathic pain [2, 3], essential tremor [4, 5], Parkinson’s disease [6, 7], and blood–brain barrier opening in patients with Alzheimer’s disease [8]. Despite the substantial benefits of tcFUS, transcranial focusing of therapeutic ultrasound waves remains a challenge because of considerable differences between acoustic properties of the skull [9], the induction of acoustic focus distortion, and focal shift, combined with a significant decrease in focal intensity in the brain tissue [10].

The solution to these limitations is the precise modulation of the amplitude and phase of transmitted acoustic waves from each element of array transducers [11]. The time-reversal approach was introduced initially for phase aberration correction; this approach relies on an implantable acoustic reflector [12] or a mono-element transducer [13, 14], and provides the optimal aberration correction as the gold-standard for validating the performance of phase correction approaches during the development stage. However, in clinical applications, an invasive insertion of the reflector or hydrophone into the brain tumor would be required, and the possibility of infection and unwanted tissue damage would be unavoidable. Another proposed approach was using a minimally invasive technique called acoustic stars [15]. A cavitation signal from a natural nucleation site [16] or an injected liquid droplet [15] was studied and the corresponding time delay was computed for aberration correction. However, the invasive placement of an acoustic source and the collapse of the droplet should be carefully controlled to avoid hemorrhage.

Among various compensation techniques, the patient’s computed tomography (CT) image-based numerical simulation provides a clinically-feasible, noninvasive aberration compensation. A full-wave acoustic model implemented on a finite-difference time-domain method based on CT image derived acoustic property provided compensation with the considerable computation cost of a few hours [17]. A fast acoustic model using a hybrid angular spectrum [18] was reported, and the computation time of 15 min was achieved with compromised accuracy. Nevertheless, a series of sonication simulations was typically planned during the tcFUS treatment to enlarge the ablation volume [5] or to treat multiple targets [19]. However, the long computation time of numerical simulation approaches is currently prohibitive from a treatment standpoint. Computation time is crucial while considering the implementation of a compensation strategy for the clinical environment. A trade-off between compensation accuracy and computation time was reported on simulation [20] and experimental [21] studies.

In an attempt to develop a lightweight phase correction toolkit, the current study focuses on the design of an opensource software implemented ray-based phase correction on a graphics process unit (GPU) of a laptop computer. An integration of developed software to the current clinical tcFUS system was carried out and three human cadaver skulls were utilized to result the phase aberration. The performance of phase correction was characterized in terms of focal point location and focal pressure based on hydrophone scanning. The refocusing performance was validated by comparing with the clinical software-based compensation in local clinical tcFUS system.

## Results

**Fig. 1:**
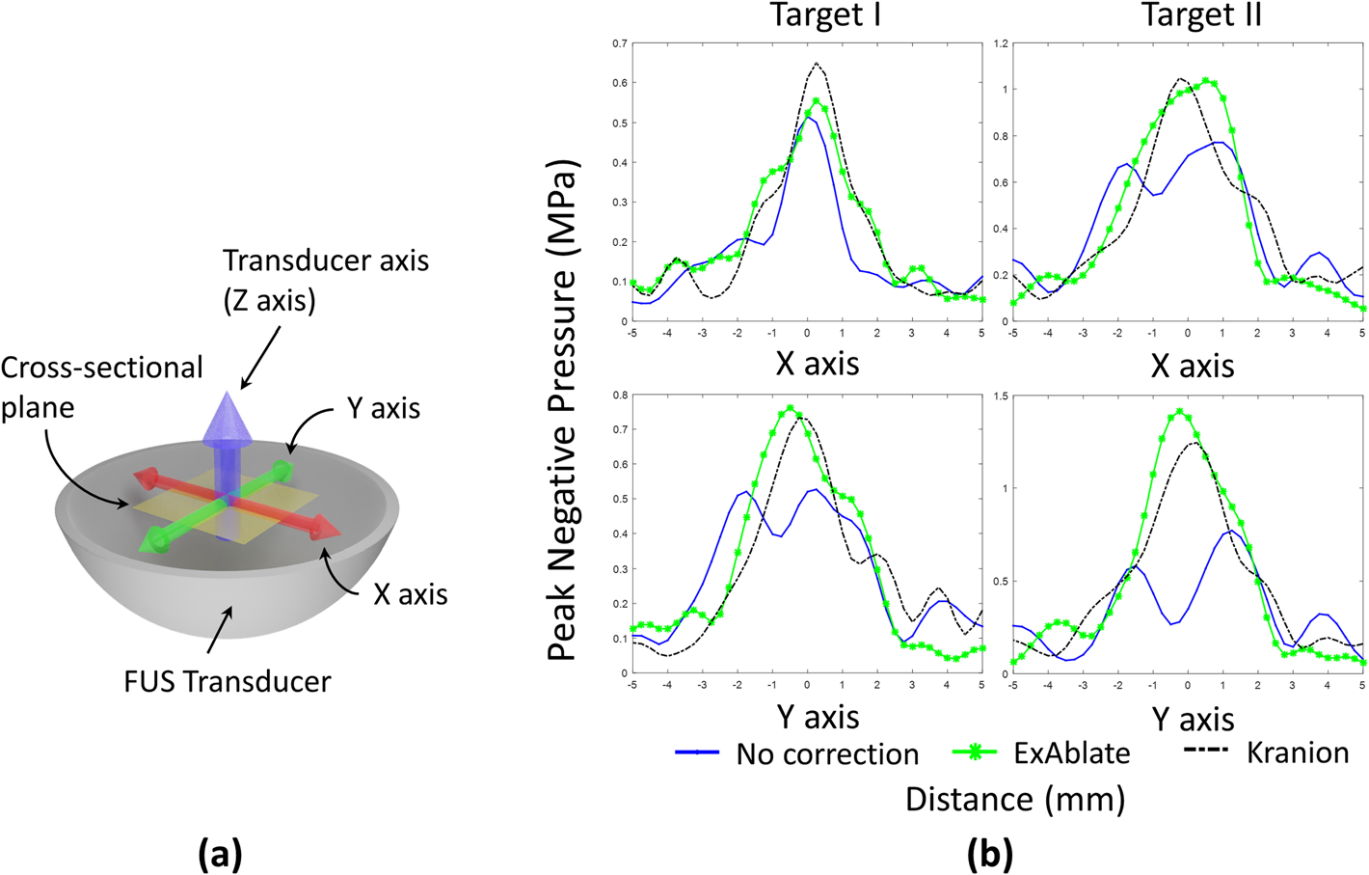
The illustration of the coordinate system and the 1D scanning results. **a** The illustration for coordinate of each axis. **b** Comparison of the one-dimensional pressure field scans of different aberration correction techniques. The measured peak negative pressure (PNP) values corresponding to the central line crossing the targeted point were collected and plotted. The results from transcranial (Skull C) focal pressure scanning without correction with ExAblate- and Kranion-based correction were illustrated. A sonication target was identified at the geometrical center of the therapeutic ultrasound transducer (Target I), and a 10 mm off-centered target was (Target II) used

The ray-based phase correction software introduced in this study was used to compute the phase correction in a sub-second speed, a total speed of 402.1 ± 24.5 milliseconds, given by the GPU’s computation capability. Two lines on the cross-sectional plane, as shown in Fig. 1a, which across the target point were scanned using hydrophone. Figure 1b exhibits the profile of the pressure field. The center of each figure was fixed to the peak point of the pre-scanned pressure map from the free field sonication. As expected, the ex vivo skull created distortion in the pressure pattern without phase correction. Kranion shows consistent compensation performance to keep the focal point on the targeted point based on these 1D pressure field scans.

**Fig. 2:**
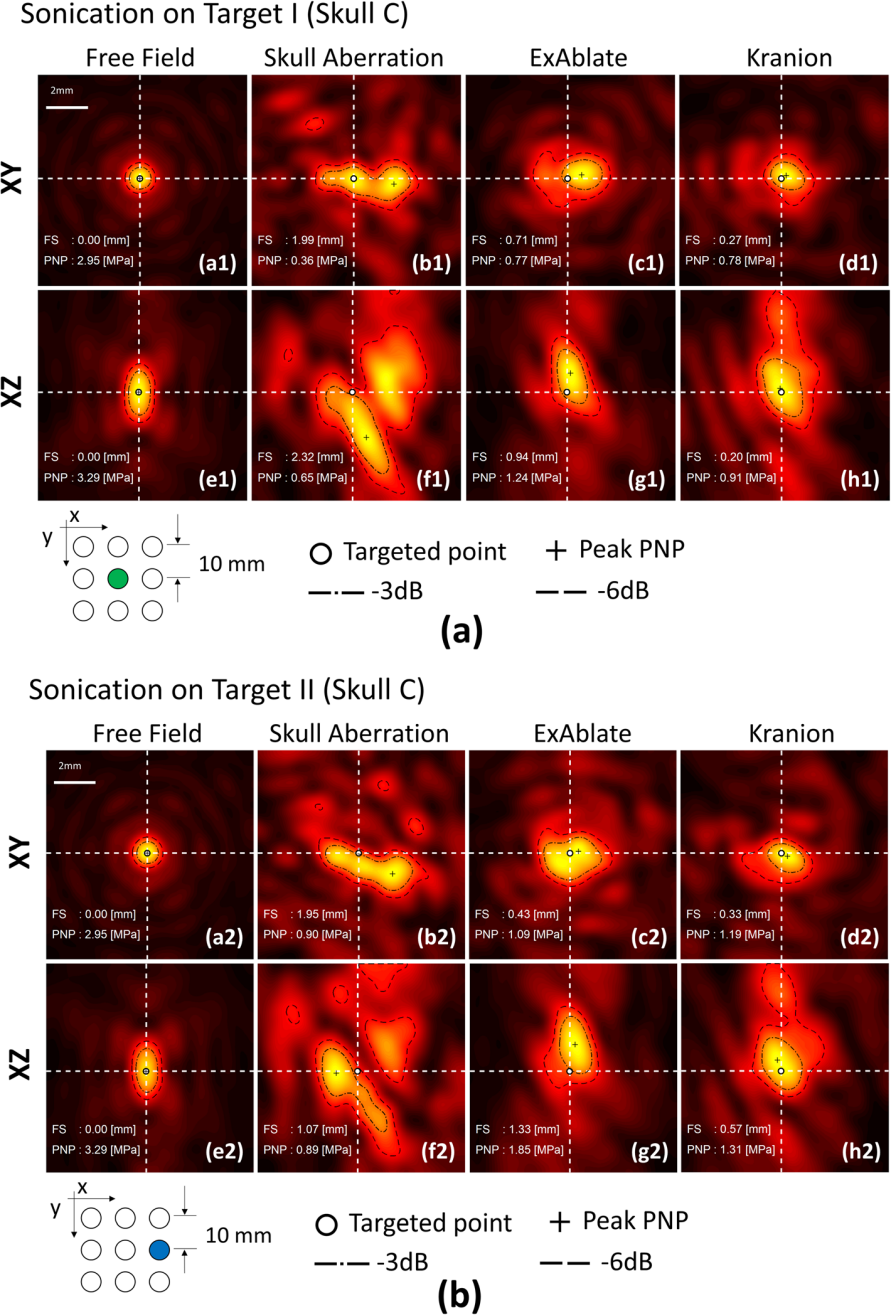
Hydrophone 2D scanning maps the human skullcap (Skull C). A 0.25 mm step resolution and 10 mm × 10 mm coverage area were maintained on all of the hydrophone scanned maps. Lateral (XY) and axial (XZ) hydrophone scanning maps based on the focal point (white dot) of the free field sonication were applied. The PNP map was plotted, and the white dashed line that crosses the focal point was illustrated. The peak intensity for each image is normalized for each local peak pixel value. The result for skull A and B were shown in additional files 1 and 2, respectively

Two orthogonal scanning planes, a cross-sectional plane (Fig. 2a1) perpendicular to the direction of the beam propagation and a longitudinal plane (Fig. 2e1) on the transducer axis, were implemented to capture the focal point in 2D. A white dot was marked on the free field sonication scan to indicate the maximum intensity point of peak negative pressure (PNP) and was utilized as a reference in the subsequent scanning and analysis. The skullcap was placed on the transducer after the focal point was localized in the scanning maps. Figure 2b1 and 6f1 present the particular aberration induced by Skull C.

**Fig. 3:**
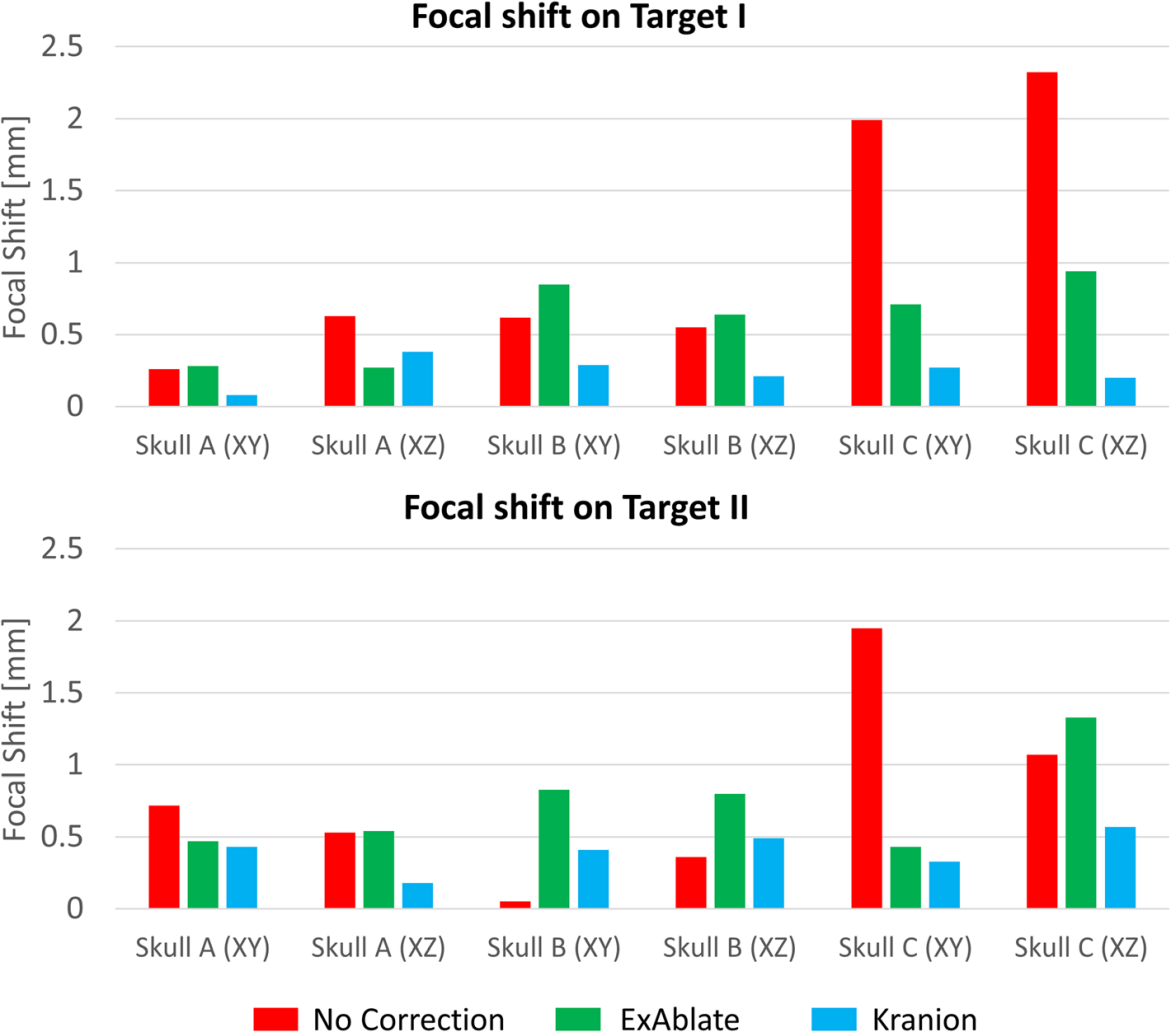
The shifted focal point on Target I and Target II. The quantified focal point shift by skull aberration and the corrected focal point by using ExAblate and Kranion method on target I and target II

The focus was spread over a large area and displaced from the intended target (Fig. 2b1, f1 and b2, f2). Two phase correction methods refocused the distorted focus. Figures 2 and 3 exhibit that Kranion demonstrated favorable performance on shifting the focal point back to the targeted point. The sonications of Target I resulted in average focal shifts of 0.44 ± 0.5, 0.34 ± 0.37, and 0.17 ± 0.15 mm for no correction, ExAblate-, and Kranion-based corrections, respectively. The sonications of Target II resulted in average focal shifts of 0.59 ± 0.44, 0.38 ± 0.34, and 0.16 ± 0.18 mm for no correction, ExAblate-, and Kranion-based corrections, correspondingly.

Table 1 lists the data depicting the peak pressure levels on the PNP map. Both correction methods improved the level of peak pressure by 152.8 ± 29.7% and 140.5 ± 17.0% on Target I and 162.6 ± 35.8% and 134.8 ± 19.1% on Target II for ExAblated and Kranion, respectively.

**Table 1:** Pressure on the focal point from 2D scanning for different correction methods

Correction Approach	Skull A		Skull B		Skull C	
	XY scan	XZ scan	XY scan	XZ scan	XY scan	XZ scan
Target I						
No Correction	0.84	1.07	0.84	0.78	0.62	0.65
ExAblate	1.53	1.68	0.99	1.13	0.77	1.24
Kranion	1.42	1.49	1.02	1.15	0.78	0.91
Target II						
No Correction	1.47	1.67	0.89	1.19	0.90	0.89
ExAblate	2.28	2.78	1.49	1.44	1.09	1.85
Kranion	2.31	2.09	1.28	1.23	1.19	1.31

## Discussion

This study aimed to evaluate a developed toolkit (Kranion) for the compensation of skull acoustic phase aberration at a clinical applicable rate by hydrophone measurements using three skull cadavers in an ExAblate 4000 system. The phase correction method introduced in this study successfully corrected the aberrated focus to the intended target position. Although the amplitude correction was not implemented in this study, a similar peak focal pressure compared to the ExAblate software-based correction was achieved. Additional improvement of focal quality could be achieved by adding an amplitude correction module [11] on the basis of transmission loss and attenuation term [22].

The local skull density, thickness, and marrow thickness were varied in different locations and directly influenced the transmission amplitude of the therapeutic wave. In particular, the inefficiency of brain tissue heating had been reported on patients with thick marrow layers [23] in their skulls. The proposed phase correction approach in this study could have enhanced compensation performance in thick marrow cases and should be addressed in future studies. Moreover, in the current MRgFUS clinical protocol, the mean skull density ratio (SDR), which is the ratio between the mean values in Hounsfield units for the marrow and cortical bone [23], is used to screen patients for treatment. Recently, a skull bone injury was reported [24], but no correlation was found between the mean SDR and the presence or absence of skull lesion. This indicated that a potential local skull heating event could be present under sufficient sonication power even with an acceptable mean SDR value. This result suggests that the per-element-based local SDR should be considered in the amplitude correction in future studies.

Table 2 lists the different numbers of activated channels from two methods. As we understand, the clinical system is designed to decide if the channel needs to be turned on or off by comparing the incident angle and critical angle. Kranion has also implemented the similar strategy to make the decision. As Table 2 shows there are different activated channel numbers between Kranion and ExAblate. We believe, the main reasons for these differences are: by 1) the difference of incident angle calculated by the image processing algorithm from two methods and 2) differences in acoustic properties, such as sound speed and density, used in two methods to calculate the critical angle. In this study, we did not attempt to mimic the exact image processing approach as that used in the clinical system software to get the same activated channel number because the technical details in the clinical system are proprietary.

**Table 2:** Activated amount of phased array channels

Correction Approach	Skull A	Skull B	Skull C
Target I			
ExAblate	982	977	967
Kranion	993	957	914
Target II			
ExAblate	981	988	883
Kranion	987	937	847

Using ray tracing algorithm to improve focal quality was notable in this study. However, this study has the following limitations. The assumptions of the transmitting plane wave from the linearized wave equation in fluid media could not account for any shear wave, which is an important mode of wave propagation in solid skull bone. This limitation could be solved by adding a shear model to the ray method [25, 26]. The nonlinear propagation phenomenon under high-intensity FUS was disregarded in this study because only low-power sonications were tested. Thus, the applicable implementation of the phase correction proposed in this study might be limited to treatments using low-intensity FUS. The number of skulls used in this study is limited to 3 full-size hemisphere skulls which is the maximum amount we could collected for this study. It is desirable to have a greater number of skulls for better validation.

The single ray representation of the acoustic beam could result in inaccurate refraction on uneven and rough skull surfaces. We observed a very few rays which hit on the non-normal voxels on the skull surface and refracted to odd directions. The implementation of a bundle of rays could filter out the influence from the roughness of the skull surface and result in an averaged ray vector.

The hydrophone-based gold-standard phase correction was desirable in validating the performance of the proposed phase correction to determine the amount of energy recovered through the method. This validation should be investigated in future studies. Two static orthogonal 2D planes were scanned to measure focal point shift which may not sufficiently represent the exact focal shift after aberration in 3D. The actual focal point could be localized more accurately by performing small 3D scans near the anticipated focus for each case and re-position the 2D measurement planes. However, it may increase the overall scanning time considerably.

The fixed value of 2900 m/s for skull bone sound speed in this study was utilized to calculate the refraction angle. The first place where sound speed value of the skull bone was utilized in proposed algorithm is refraction angle calculation (5) which is right after the collision point detection. In this stage, the algorithm doesn’t have any knowledge about the skull bone sound speed to calculate the refraction angle. In order to apply the Snell’s law, we utilized the representative sound speed as an initial value for skull bone. Nevertheless, the heterogeneous property of the skull bone was accounted in later stage of the algorithm to calculate the aberrated phase (6).

## Conclusions

A ray-based lightweight toolkit to compensate aberrated phase was developed. The performance of the toolkit was validated by hydrophone measurements through three skull cadavers in a clinical configuration. The accuracy and computation time of the proposed phase correction method are reasonable for its usage in pre-clinical transcranial focused ultrasound research.

## Methods

### Ray-skull collision detection

**Fig. 4:**
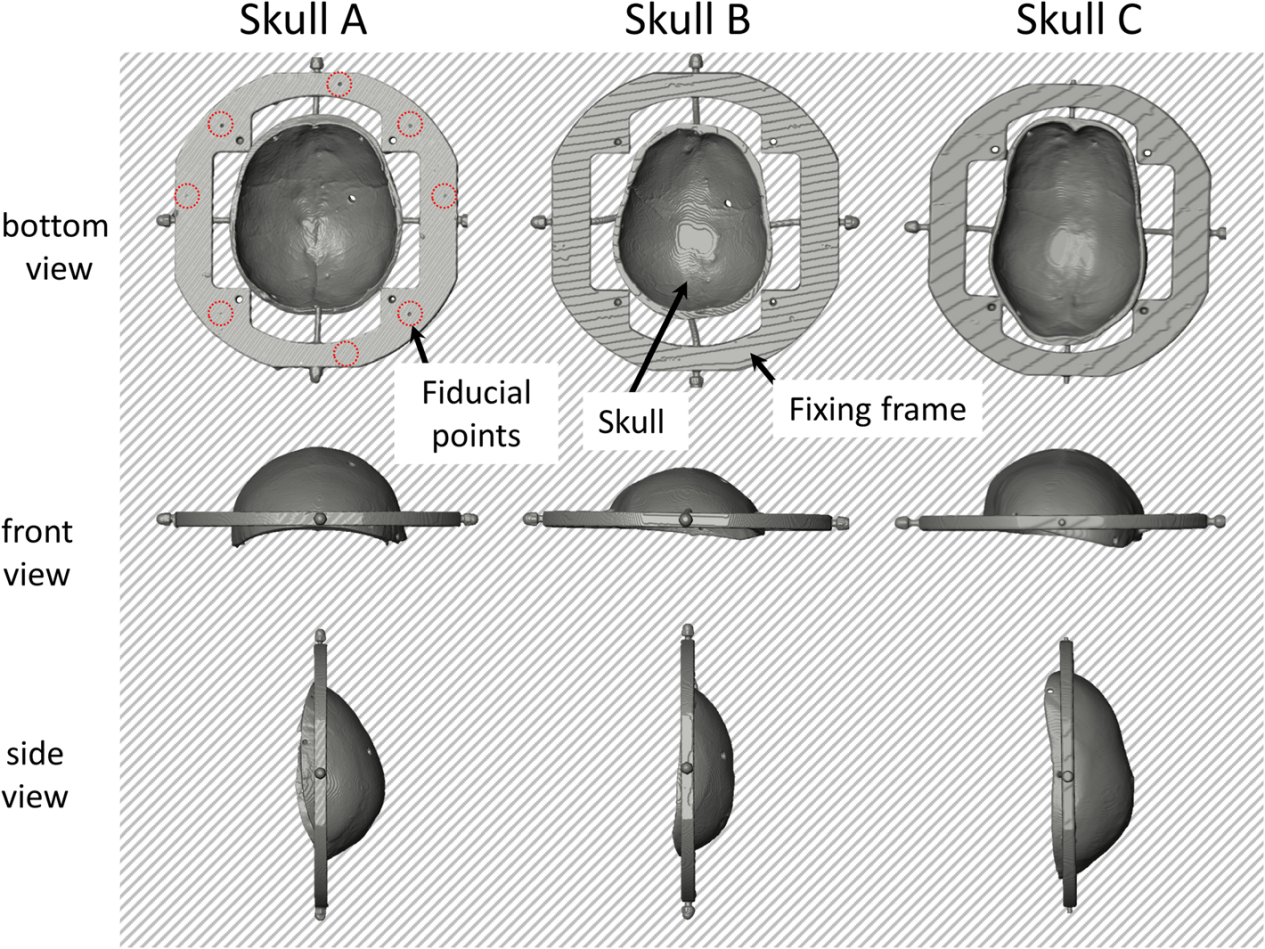
Reconstructed volume rendering of three skulls with their fixing frame. The volume of skull and fixing frame were reconstructed from CT volume images. The bottom, front and side view of three skull volume were shown in the figure. The visible fiducial points of skull A (seven points) were marked using red-doted circles. The variation of shape and surface curvature of each skull could be observed

While an acoustic wave emitted from an ultrasound transducer element propagates along a beam in a medium, it reaches the boundary of adjacent medium. In transcranial focused ultrasound, the intersection points between the wave beam and the boundaries of different medium, such as water, skull bone, and brain tissue, should be defined to implement ray tracing. A threshold-based collision point detection was implemented on the pre-scanned CT volume images of the cadaver skull to identify the coordinates of the intersection points. The volume rendering of three cadaver skulls with fixing frame was reconstructed from scanned CT image as shown in Fig. 4.

**Fig. 5:**
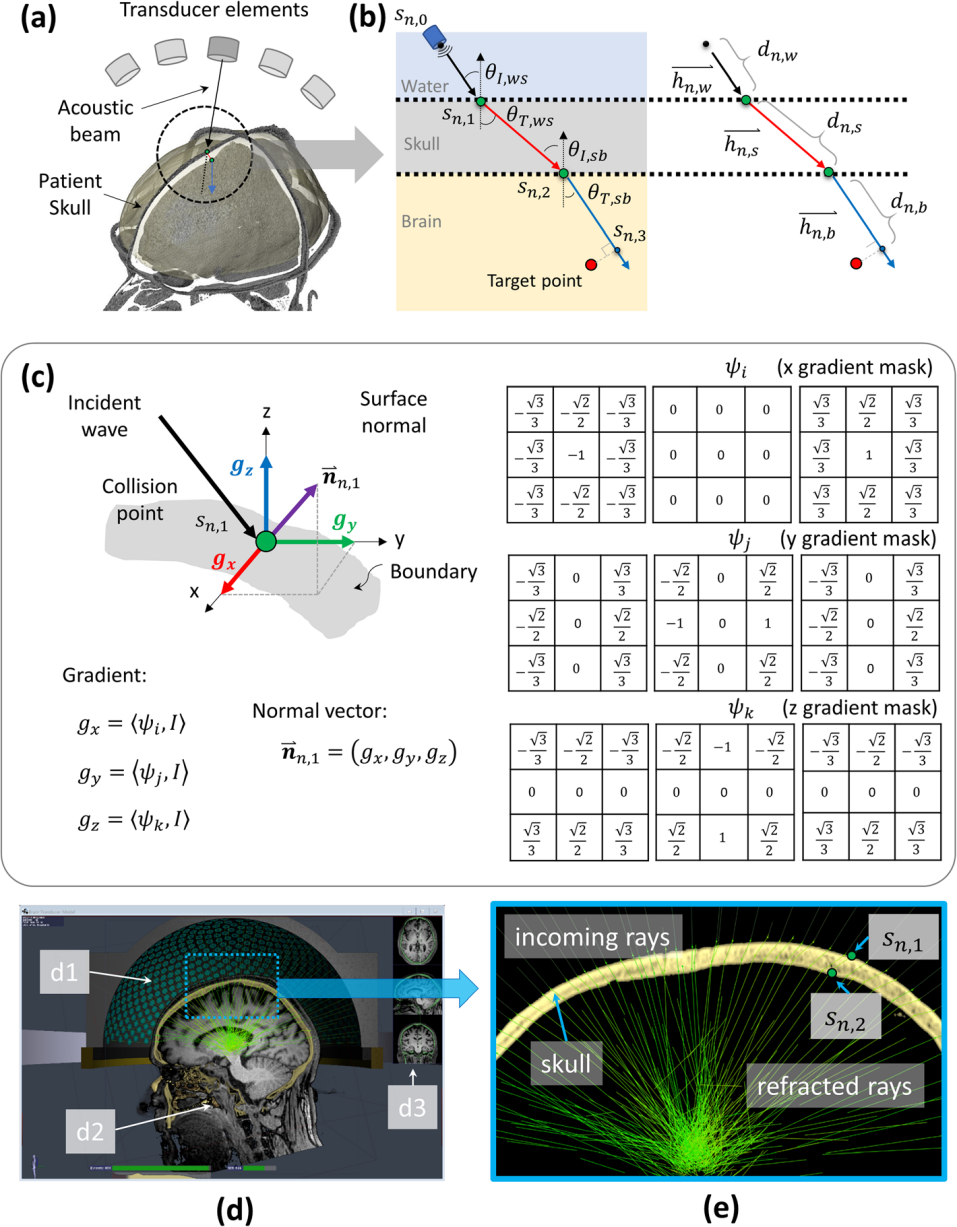
Overview of proposed ray-tracing based phase correction. **a** Some of the 1024 elements of the clinical focused ultrasound (FUS) transducer and reconstructed 3D patient head based on computed tomography (CT) images. A threshold-based collision point detection was applied to define the intersection point between incident vector and skull. **b** The refraction diagrams on water-to-skull and skull-to-brain boundary. **c** The edge operator on three axis and the equations to estimate the normal vector of the skull surface on the basis of the defined collision point. **d** Overview of developed graphic user interface (GUI) of the Kranion software. Illustrating the FUS transducer (d1) and magnetic resonance imaging (MRI)–CT registration (d2 and d3) of the same patient. **e** Zoomed view of the blue rectangular depicted in (**d**). The refracted rays (green lines) on *s*_*n*, 1_ and *s*_*n*, 2_ of 1024 channels were rendered in a sub-second speed. Note that the MRI image was not utilized in phase correction experiment. The MRI image shown in this figure is to show the MRI-CT image registration capability of developed software

The central coordinate of 1024 elements (*n*) in a clinical focused ultrasound system, *s*_*n*, 0_, was obtained from the system manufacturer. The notation *n* represents the element number of the array transducer and 0 is the indicator to represent the transducer coordinate. Similarly, the number 1 and 2 were indicated the coordinate of collision points on water-to-skull boundary and skull-to-brain boundary, respectively. A geometric-center-oriented vector $\overset{\rightharpoonup }{h_{n,w}}$ represents the vector of the emitted wave illustrated as a black arrow in Fig. 5a. The ray was traversed with a step size of 0.1 mm. The end point of the vector is, which is the first collision point, defined as below:

1 $$ {s}_{n,1}={s}_{n,0}+0.1\times {d}_{n,w}\times \overset{\rightharpoonup }{h_{n,w}.} $$

In order to search the collision point, the corresponding trilinear interpolated voxel value *Q*_*n*, 1_ on the point *s*_*n*, 1_ was collected and compared with the predefined Hounsfield Unit threshold *τ*_*skull*_ = 700. The integer increments *d*_*n*, *w*_ iteratively increase its value by 1 until the following condition was met. The first collision point, *s*_*n*, 1_, was localized by searching the end point which satisfy *Q*_*n*, 1_ ≥ *τ*_*skull*_. Then the normal vector of the water-skull boundary was calculated based on localized collision point. The incident and refraction angles were calculated after the normal vector was defined. The details about incident angle, refraction angle and normal vector calculation were explained in following sections.

The second collision point, *s*_*n*, 2_, was defined by following the same detection method of the first collision point. However, the end point which satisfy *Q*_*n*, 2_ ≤ *τ*_*skull*_ was searched and defined as the second collision point. The cancellous bone, which has lower Hounsfield unit (HU) value than the threshold, was simply neglected by defining two computation regions based on two HU peaks, inner and outer cortical bones on collected HU profile along the corresponding ray segment.

### Surface normal estimation

A normal vector of the skull surface that occurs on the ray–skull collision point is required to compute the incident and refraction angles of the wave beam (Fig. 5b). The edge of the surface is a small area in the image volume, where the local level changes rapidly. Thus, an edge operator was utilized to detect the best-oriented plane at estimated collision points.

The mathematical foundation for obtaining the three basic functions that estimate the local image gradient was introduced by Zucker and Hummel [27, 28]. A set of three 3x3x3 operators (*ψ*_*i*_, *ψ*_*j*_ and *ψ*_*k*_) are used to estimate the image gradient at any voxel of interest, each providing the directional gradient value in x, y and z directions respectively.

2 $$ {\displaystyle \begin{array}{c}{\psi}_i\left(x,y,z\right)=x/\sqrt{x^2+{y}^2+{z}^2}\\ {}{\psi}_j\left(x,y,z\right)=y/\sqrt{x^2+{y}^2+{z}^2}\\ {}{\psi}_k\left(x,y,z\right)=z/\sqrt{x^2+{y}^2+{z}^2\ }\end{array}} $$

The discrete approximation of these three operators were shown in Fig. 5c. Then the surface normal defining the best edge at collision point, i.e. (α, β, γ), was obtained by convolving *ψ* with the input image I.

3 $$ {\displaystyle \begin{array}{c}{g}_x=\left\langle {\psi}_i,I\right\rangle ={\iiint}_{\delta }{\psi}_i\left(x,y,z\right)I\left(\mathrm{x}-\alpha, y-\beta, z-\gamma \right) dx\  dy\  dz\\ {}{g}_y=\left\langle {\psi}_j,I\right\rangle \\ {}{g}_z=\left\langle {\psi}_k,I\right\rangle \end{array}} $$

Where *g*_*x*_, *g*_*y*_ and *g*_*z*_ are gradient component along x, y and z axis, respectively. Finally, the normal vector on the skull surface based on the detected collision point is defined as

4 $$ {\overset{\rightharpoonup }{\boldsymbol{n}}}_{n,1}=\left({g}_x,{g}_y,{g}_z\right) $$

### Refraction-based aberration phase

The incident angle *θ*_*I*, *ws*_ can be calculated on the basis of the detected normal vector ${\overset{\rightharpoonup }{\boldsymbol{n}}}_{n,1}$ and the vector $\overset{\rightharpoonup }{h_{n,w}}$ of incident wave (see Fig. 5b). Then, the refraction angle *θ*_*T*, *ws*_ could be derived based on Snell’s Law as shown in bellow:

5 $$ \frac{\mathit{\sin}{\theta}_{I, ws}}{C_w}=\frac{\mathit{\sin}{\theta}_{T, ws}}{C_s} $$

, where *θ*_*I*, *ws*_ and *θ*_*T*, *ws*_ denote the incident angle and refraction angle on the water-to-skull boundary, respectively. And *c*_*w*_ and *c*_*s*_ are the averaged sound speed of water and skull, respectively. The sound speed for water and skull was defined as 1480 m/s and 2900 m/s, respectively [29]. Then, the refracted vector $\overset{\rightharpoonup }{h_{n,s}}$ that travels inside of the skull bone could be defined. The second collision point was searched based on the above-mentioned method and the normal vector ${\overset{\rightharpoonup }{\boldsymbol{n}}}_{n,2}$ and incident angle *θ*_*I*, *sb*_ were calculated accordingly. Then, the refraction angle *θ*_*T*, *sb*_ and refracted vector $\overset{\rightharpoonup }{h_{n,b}}$ could be defined based on Snell’s Law.

6 $$ \frac{\mathit{\sin}{\theta}_{I, sb}}{C_s}=\frac{\mathit{\sin}{\theta}_{T, sb}}{C_b} $$

, where *c*_*b*_ denotes the sound speed of brain region and it was set to be the same as the water in this study, because ex-vivo experiment was performed by merging skull into the water without any brain tissue or phantom placed inside the skull.

The length of the ray segments from transducer to first collision point *d*_*n*, *w*_, traverses the skull *d*_*n*, *s*_ and the length from second collision point to the point that has the closest distance to targeted point *d*_*n*, *b*_ were collected for each transducer element. The aberrated phase was then calculated using the following equation:

7 $$ {\varnothing}_n=2\pi {f}_0\left(\frac{d_{n,w}}{c_{n,w}}+\frac{d_{n,s}}{c_{n,s}}+\frac{d_{n,b}}{c_{n,b}}\right) $$

, where *f*_0_ is the driving frequency of the FUS system (650 kHz), and *c*_*n*, *w*_, *c*_*n*, *s*_ and *c*_*n*, *b*_ are the averaged sound wave speeds which were estimated by averaging the sound speed derived from the Hounsfield unit in the CT image [30] along each segment of the ray. Note that the wave speed in brain medium *c*_*n*, *b*_ was defined as equal as the wave speed in water *c*_*n*, *w*_ because no brain tissue was utilized in the experiment. The aberrated phase ∅_*n*_ of each element *n* was obtained, and additional phase unwrapping to define actual excitation phase range (as – *π* to *π*) for FUS transducer was performed. Phases were then printed out as a phase correction configuration file to be imported into the control computer (CPC) of the clinical system. Notably, incident angles that are greater than the critical angle (total reflection) were neglected in the phase computation, and the corresponding elements were turned off during the experiment.

### Kranion software

By implementing the aforementioned technique, Kranion software was developed by the authors in [31], and Fig. 5d exhibits a screen capture of the GUI. Figure 5e illustrates a close-up view of the refracted rays. The software was developed using the Java programing language on the NetBeans integrated development environment (Apache Software Foundation, https://netbeans.apache.org) and available for free download from GitHub (https://github.com/jws2f/Kranion) under MIT license. The main ray tracing computation was constructed as a compute shader executing on a GPU (GeForce GTX 1080 with 12 GB RAM, NVIDIA, CA, US). The software allows a user to manually register the MR and CT images and rotate the scene to any desired observation view. The FUS transducer geometry could be moved to an arbitrary position, and the corresponding ray tracing of 1024 elements was rendered in a sub-second speed and illustrated on the screen of a laptop computer. Additional information on the software is available in [31].

### Registration

**Fig. 6:**
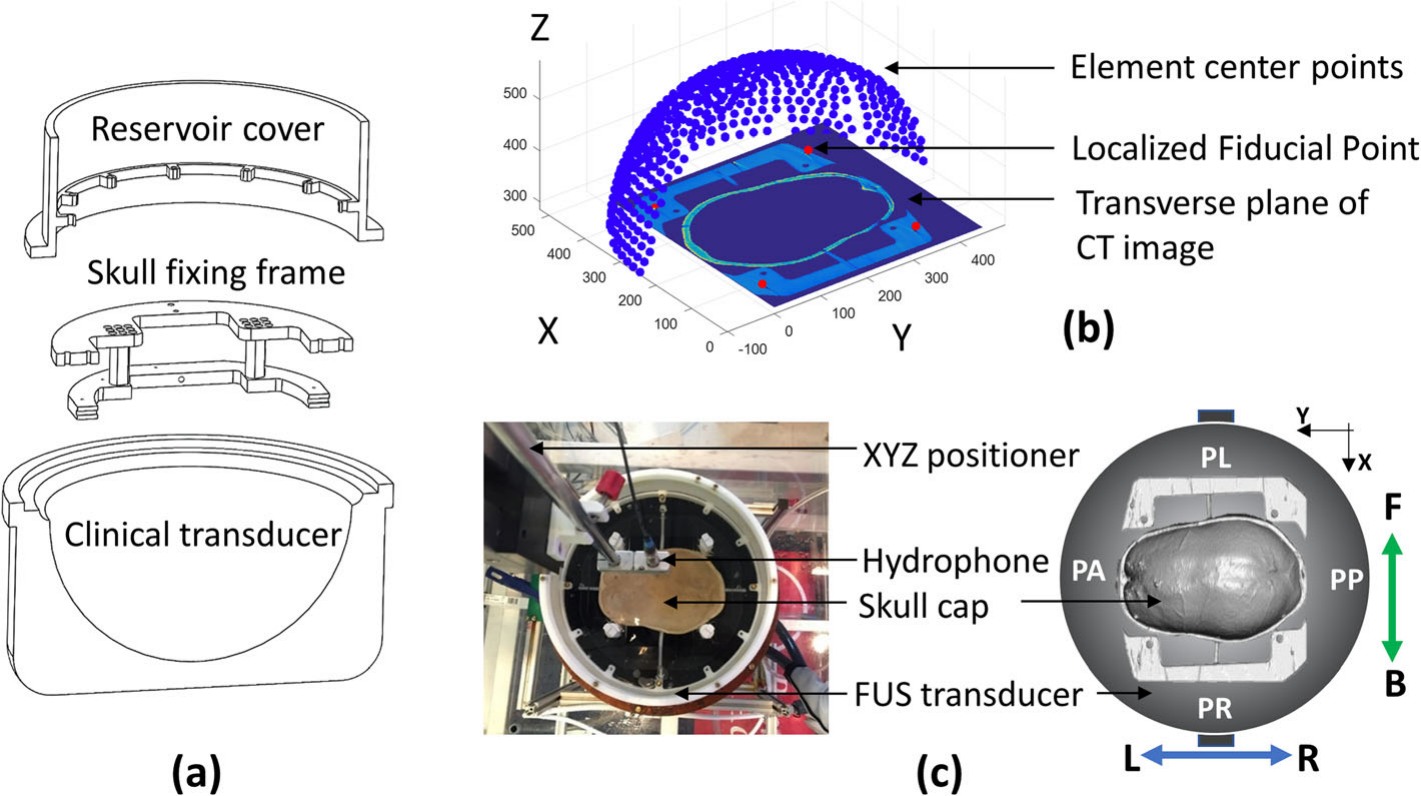
The registration of clinical focused ultrasound system and cadaver skull. **a** Computer-aided design (CAD) sketch of the clinical transducer, skull fixing frame, and reservoir cover. **b** Fiducial point-based registration of the transducer elements and skull volume in scanned CT images. Notably, the detailed geometry of the actual FUS transducer was excluded in this study. The sketch in (**a**) and (**b**) does not represent the actual scale of the FUS transducer. **c** Top view illustrating the coordinate configuration, including patient perspective coordinate (patient’s anterior-PA & patient’s posterior-PP, patient’s left-PL & patient’s right-PR), CT image coordinate, and coordinate of hydrophone scanning system (front [F] & back [B], left [L] & right [R])

To simulate the skull phase aberration, the registration between transducer elements and the cadaver skull was obtained by using fiducial points through computer-aided design (CAD) fabrication and CT scanning. A cover and skull fixing frame were fabricated on the basis of the CAD sketch and assembled on top of the transducer. Figure 6a depicts a simplified sketch. Eight tantalum fiducial points (2 mm diameter, Bal-Tec, CA, US) were embedded in the known coordinates of the fixing frame during fabrication. The registration between the transducer and fiducial points was defined using an absolute geometry of the transducer and skull fixing frame. The coordinates of the fiducial points *FP*_*Transducer*_ based on the transducer element coordinate system were then derived. Three cadaver skullcaps were obtained (University Hospital of Virginia, VA, US) and fastened on the fixing frame. A 3D volume image of the assembled skull frames was obtained using the LightSpeed VCT CT system (GE Healthcare, IL, US). The coordinates of the fiducial points *FP*_*CT*_ were defined by binarizing the CT image of each skull using an appropriate threshold. Granulometry was used to identify the fiducial regions, and the center of mass was calculated for each region to obtain the fiducial position.

The transformation function between the transducer coordinate system and CT volume was then calculated using singular-value decomposition (SVD) method [32]. The *FP*_*Transducer*_ can be represented as

8 $$ SVD\left({FP}_{Transducer}\right)= U\varSigma {V}^{\ast }, $$

where *U* (m × m) and *V* (n × n) are the unitary matrices, and *Σ* is the diagonal matrix (m × n). *V*^∗^ is the conjugate transpose of *V*. Then, the rotation matrix *R* is expressed as

9 $$ R=U{V}^{\ast } $$

and the translation matrix *T* is defined as

10 $$ T=-R\bullet {FP}_{Transducer}+{FP}_{CT} $$

The final transformation between the voxel center in the CT image (*P*_*CT*_) and the transducer coordinate system (*P*_*Transducer*_) can be derived using

11 $$ {P}_{CT}=R\bullet {P}_{Transducer}+T $$

Figure 6b plots the composite figure of the CT skull frame image and the transducer elements. To obtain an improved see-through view, only half of the transducer is shown. Figure 6c depicts the coordinate configuration lookup table between the software and experiment hardware systems. This configuration combines the coordinate systems of the transducer, patient, and hydrophone scanning domain.

### Experimental setup and hydrophone scanning

The experiment was implemented on the magnetic imaging guided focused ultrasound (MRgFUS) clinical system at the Focused Ultrasound Center of the University of Virginia (University of Virginia Health System, VA, US). An ExAblate 650 kHz FUS transducer was utilized in this study. The transducer was carefully detached from the treatment bed and placed in an AIMS III scanning tank (AST3-L, ONDA, CA, US). The facing up transducer was mounted on a constructed frame that was fixed inside the scanning tank (Fig. 6c). The transducer filled with degassed water was positioned in an empty scanning tank during the whole experiment. The degassed water (with the remaining oxygen level maintained at less than 1.1 ppm) was prepared using collected tap water and a vacuum membrane degassing system before configuring the experimental setup. Skull degassing was performed for 1 h before attaching the skull frame to the FUS transducer.

The clinical brain transducer consists of 1024 elements, and each element is individually driven on the basis of predefined phase correction files. The format of the phase correction file that is compatible with ExAblate console software was studied and a new configuration file was reconstructed in accordance with the proposed phase correction calculated by the Kranion software. The Kranion- and ExAblate-based phase correction files were fed into the CPC, and the corresponding sonications were scheduled to compare the resulting pressure fields.

**Fig. 7:**
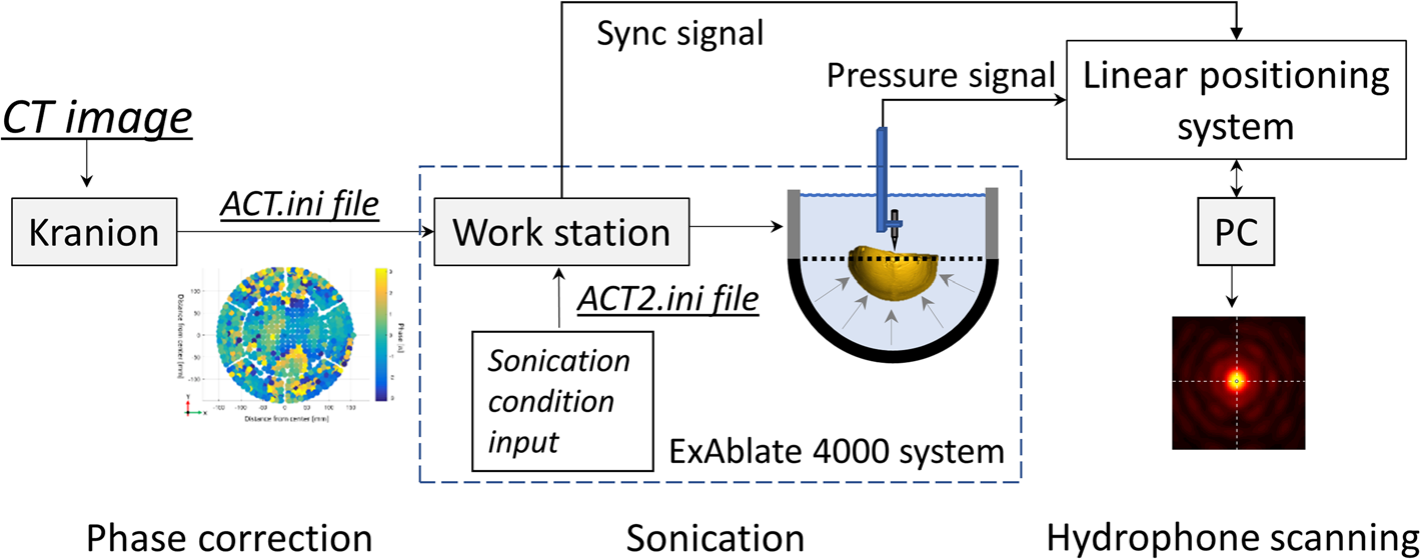
The diagram of experiment workflow. **a** The schematic setup for hydrophone scanning experiment. The phase correction file was constructed in the Kranion software on the basis of the pre-scanned CT image of each collected cadaver skull. The software in the workstation of the clinical system was scheduled for phase-corrected sonication on the basis of the imported correction file. A series of hydrophone scanning was performed on the focal plane to map the transcranial pressure field. The 2D pressure field data of each scanning were collected for postprocessing

Figure 7 demonstrates an overview of the experimental setup. A needle-type hydrophone (HNA-0400, ONDA, CA, US) and a preamplifier (AH2020, ONDA, CA, US) were utilized to measure the pressure variation in the region of interest. The amplified pressure signal was obtained using an oscilloscope that was synchronized to the transducer driving system through a Bayonet Neill-Concelman connector. The raw data of each 2D pressure scan were saved and labeled in the PC that controlled the hydrophone positioning system.

## Supplementary information

**Additional file 1.** Hydrophone 2D scanning maps of the free field sonication, skull aberration without correction, with ExAblated- and Kranion-based corrections on the human skullcap (Skull A). A 0.25 mm step resolution and 10 mm × 10 mm coverage area were maintained on all of the hydrophone scanned maps. Lateral (XY) and axial (XZ) hydrophone scanning maps based on the focal point (white dot) of the free field sonication were applied. The PNP map was plotted, and the white dashed line that crosses the focal point was illustrated. The peak intensity for each image is normalized for each local peak pixel value.

**Additional file 2.** Hydrophone 2D scanning maps of the free field sonication, skull aberration without correction, with ExAblated- and Kranion-based corrections on the human skullcap (Skull B). A 0.25 mm step resolution and 10 mm × 10 mm coverage area were maintained on all of the hydrophone scanned maps. Lateral (XY) and axial (XZ) hydrophone scanning maps based on the focal point (white dot) of the free field sonication were applied. The PNP map was plotted, and the white dashed line that crosses the focal point was illustrated. The peak intensity for each image is normalized for each local peak pixel value.

## Data Availability

The opensource toolkit in this paper is available from https://www.fusfoundation.org/for-researchers/resources/kranion and the datasets used and/or analyzed during the current study are available from the corresponding author on reasonable request.

## Abbreviations

tcFUS: Transcranial focused ultrasound
CT: Computed tomography
GPU: Graphics process unit
FUS: Focused ultrasound
GUI: Graphic user interface
MRI: Magnetic resonance imaging
HU: Hounsfield unit
CPC: Control computer
CAD: Computer aided design
SVD: Singular-value decomposition
MRgFUS: Magnetic imaging guided focused ultrasound
PNP: Peak negative pressure
SDR: Skull density ratio
