# Supplementary figures and images for: An open-source phase correction toolkit for transcranial focused ultrasound
Source: BMC Biomed Eng. 2020 Aug 14;2:9. doi: 10.1186/s42490-020-00043-3 (PMC7427913; doi:10.1186/s42490-020-00043-3)

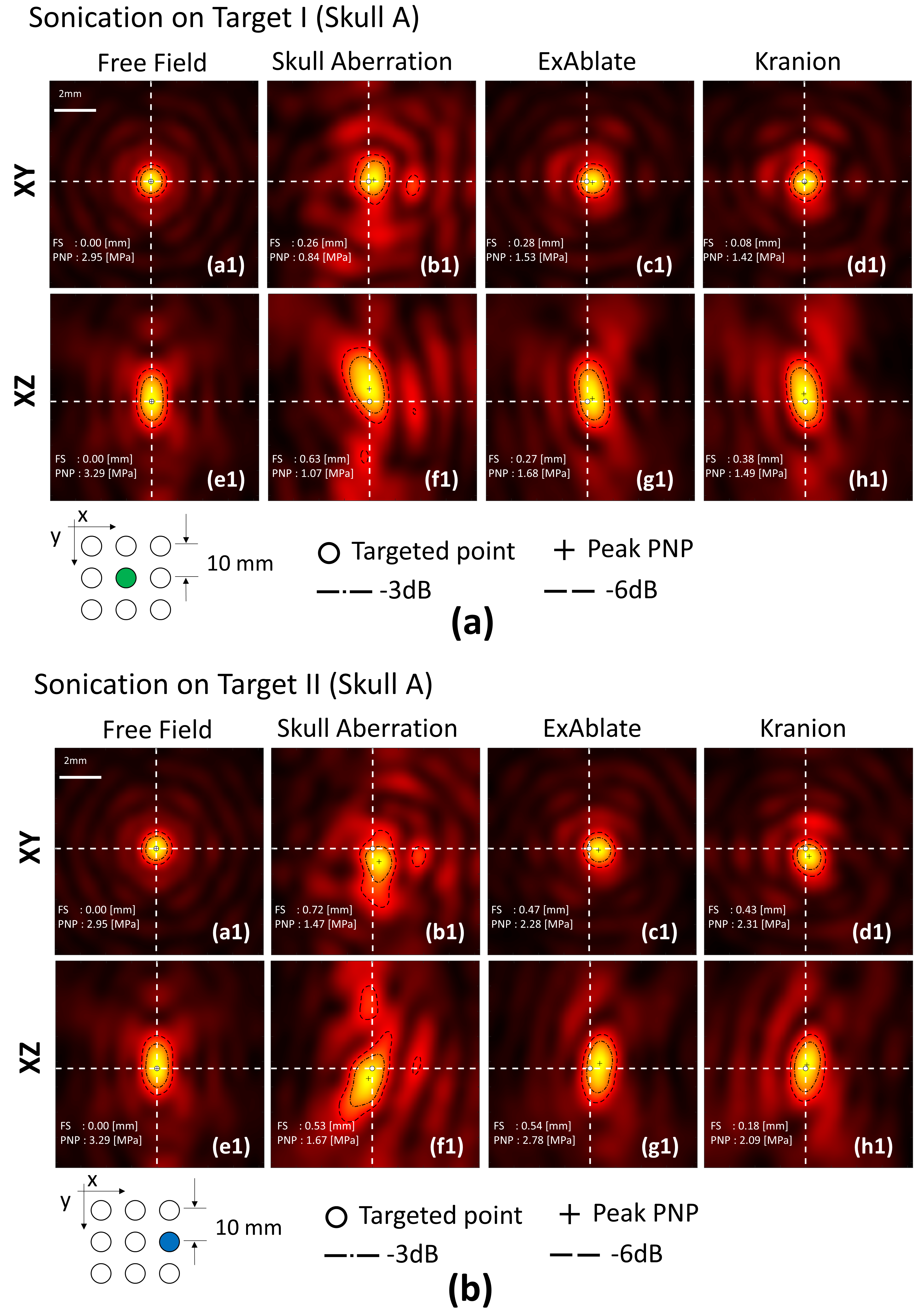

Supplement: Supplementary file 1 — Additional file 1. Hydrophone 2D scanning maps of the free field sonication, skull aberration without correction, with ExAblated- and Kranion-based corrections on the human skullcap (Skull A). A 0.25 mm step resolution and 10 mm × 10 mm coverage area were maintained on all of the hydrophone scanned maps. Lateral (XY) and axial (XZ) hydrophone scanning maps based on the focal point (white dot) of the free field sonication were applied. The PNP map was plotted, and the white dashed line that crosses the focal point was illustrated. The peak intensity for each image is normalized for each local peak pixel value. [file 42490_2020_43_MOESM1_ESM.tif]

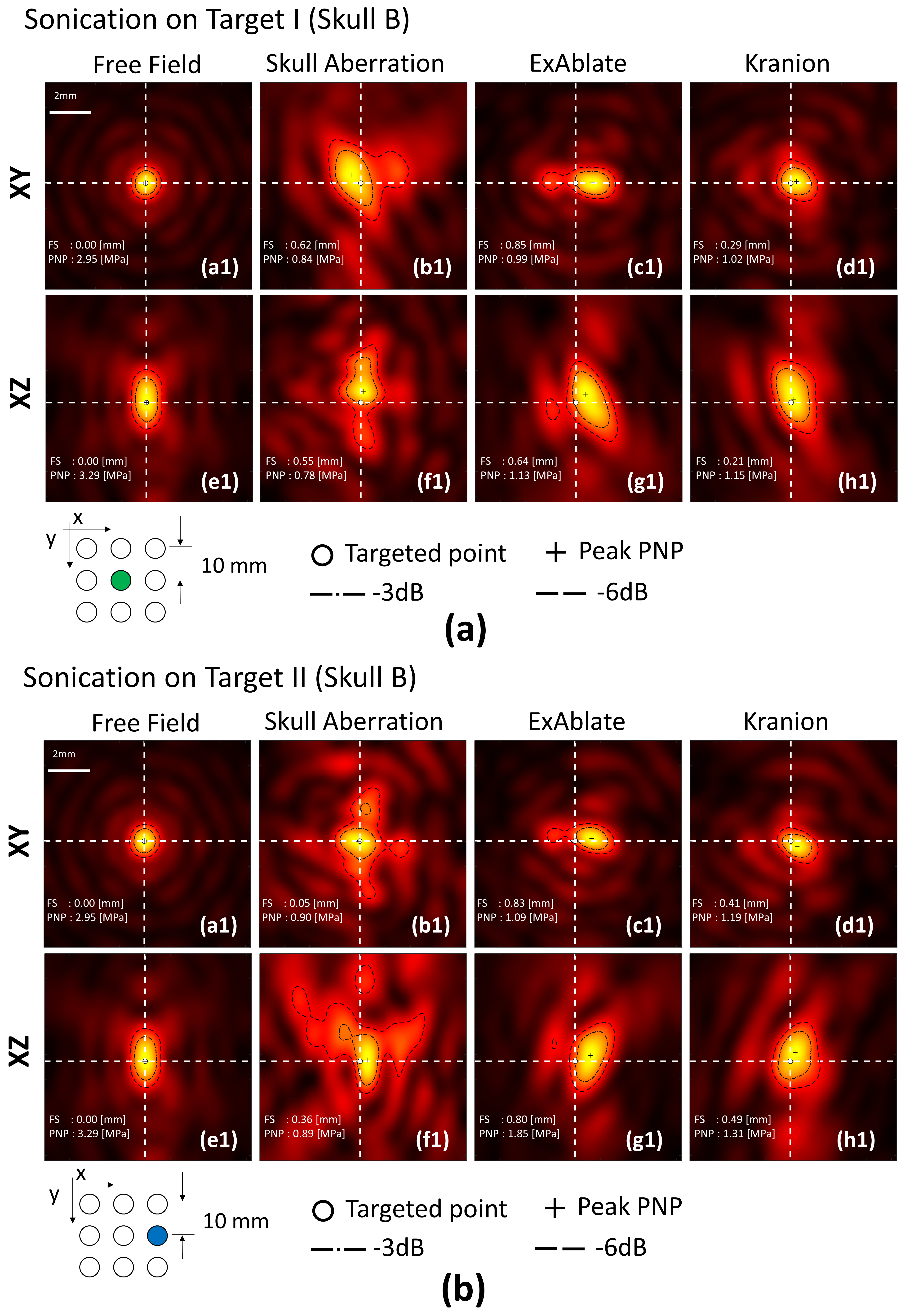

Supplement: Supplementary file 2 — Additional file 2. Hydrophone 2D scanning maps of the free field sonication, skull aberration without correction, with ExAblated- and Kranion-based corrections on the human skullcap (Skull B). A 0.25 mm step resolution and 10 mm × 10 mm coverage area were maintained on all of the hydrophone scanned maps. Lateral (XY) and axial (XZ) hydrophone scanning maps based on the focal point (white dot) of the free field sonication were applied. The PNP map was plotted, and the white dashed line that crosses the focal point was illustrated. The peak intensity for each image is normalized for each local peak pixel value. [file 42490_2020_43_MOESM2_ESM.tif]
